# Prevalence of anti-hepatitis E virus IgG antibodies in sera from hemodialysis patients in Tripoli, Lebanon

**DOI:** 10.1371/journal.pone.0233256

**Published:** 2020-05-18

**Authors:** Mohamad Bachar Ismail, Imad Al Kassaa, Dima El Safadi, Sarah Al Omari, Hassan Mallat, Fouad Dabboussi, Monzer Hamze

**Affiliations:** 1 Laboratoire Microbiologie Santé et Environnement (LMSE), Doctoral School of Sciences and Technology, Faculty of Public Health, Lebanese University, Tripoli, Lebanon; 2 Faculty of Science, Lebanese University, Tripoli, Lebanon; 3 Faculty of Public Health, Lebanese University, Tripoli, Lebanon; Centers for Disease Control and Prevention, UNITED STATES

## Abstract

Hepatitis E virus (HEV) is an important global public health concern. Several studies reported a higher HEV prevalence in patients undergoing regular hemodialysis (HD). In Lebanon, the epidemiology of HEV among HD patients has never been investigated previously. In this study, we examine the seroprevalence of HEV infection among 171 HD patients recruited from three hospital dialysis units in Tripoli, North Lebanon. Prevalence of anti-HEV IgG antibodies was evaluated in participant’s sera using a commercial enzyme-linked immunosorbent assay (ELISA). The association of socio-demographic and clinical parameters with HEV infection in patients was also evaluated. Overall, 96 women and 75 men were enrolled in this study. Anti-HEV IgG antibodies were found positive in 37/171 HD patients showing a positivity rate of 21.63%. Among all examined variables, only the age of patients was significantly associated with seropositivity (P = 0.001). This first epidemiological study reveals a high seroprevalence of HEV infection among Lebanese HD patients. However, further evaluations that enroll larger samples and include control groups are required to identify exact causative factors of the important seropositivity rate in this population.

## Introduction

Hepatitis E virus (HEV), is a small, non-enveloped, positive-sense single-stranded RNA virus that is globally distributed [1]. This virus is a significant cause of morbidity and mortality and represents an important global health concern. The World Health Organization (WHO) estimates that every year there are 20 million new HEV infections resulting in 3.3 million acute cases and nearly 44 000 related-deaths in the world [2].

Clinical manifestation of HEV infection shows a large spectrum of outcomes ranging from asymptomatic and subclinical infections to life-threatening fulminant hepatic failure. Infection may also progress to a chronic course, especially in immunocompromised patients, and may occasionally cause extra-hepatic disorders [3].

A variety of human and animal HEV strains exist and show a significant heterogeneity level as a result of very complex evolution conditions [4]. Indeed, HEV belongs to the *Hepeviridae* family which is currently divided into two genera: *Orthohepevirus* (containing all mammalian and avian isolates) and *Piscihepevirus* (trout isolate) [5]. The genus *Orthohepevirus* is divided into A-D at the species level while the genus *Piscihepevirus* contains only one species called *Piscihepevirus A*. *Orthohepevirus* B-D and *Piscihepevirus A* consist of HEV strains that infect animals but not humans. In contrast, *Orthohepevirus A*, which contains 8 genotypes, includes HEV strains that are transmissible to humans. These latter comprise genotypes 1, 2, 3, 4, and, more recently, 7 [6].

Two distinct epidemiological patterns were classically reported for HEV-induced human infections. HEV genotypes 1 and 2, only found in humans, are highly prevalent in developing countries and cause epidemic hepatitis associated with waterborne and fecal-oral transmission. In contrast, HEV genotypes 3, 4 and 7 are zoonotically transmitted from animal reservoirs and the former two genotypes are essentially responsible for sporadic autochthonous cases in developed countries [6–9]. However, independent reports showed that HEV transmission may also occur through blood transfusion [10] and from patient-to-patient through nosocomial transmission [11]. In this context, several studies reported a high prevalence of anti-HEV antibodies among hemodialysis (HD) patients suggesting that this population represents a high-risk group for this viral infection [12,13]. Nevertheless, others suggested that chronic HD is not associated with an increased risk of exposure to this virus [14,15].

In Lebanon, epidemiological data concerning HEV circulation in the country are very scarce and the national prevalence of this virus among HD patients has never been previously explored. In the present study, we investigated the current prevalence and associated risk factors of HEV infection in this population in Tripoli, North Lebanon.

## Materials and methods

### Patients and data collection

This descriptive cross-sectional study was carried out in Tripoli, the largest city in the North of Lebanon and the second Lebanese city after the capital Beirut, during July 2016. It includes a total of 171 patients undergoing regular HD in three different hospital HD units: Al Nini (n = 69), Orange Nassau (n = 49) and Al Islami (n = 53). Socio-demographic data of each patient were collected using a questionnaire survey listing their age, sex, educational level and source of drinking water. Patient’s medical records were also used to collect information concerning the onset of starting, frequency and duration of HD as well as possible infections by hepatitis B and C viruses (HBV and HCV). HBV infection was diagnosed by the detection of hepatitis B surface antigen (HBs Ag) using Architect HBsAg assay (Abbott), Vidas HBsAg Ultra (bioMérieux), and ADVIA Centaur HBsAg (Siemens) at Al Nini, Orange Nassau and Al Islami hospitals, respectively. In a similar respective order, infection by HCV was diagnosed by the detection of anti HCV antibodies using Architect anti-HCV assay (IgG/IgM, Abbott), VIDAS anti-HCV assay (IgG, bioMérieux) and ADVIA Centaur HCV assay (IgG, Siemens).

### Ethics statement

This study was approved by the ethics committee of the Azm Center for Research in Biotechnology and Its Applications, Lebanese University (approval no. CE-EDST-2-2015). Written informed consent was obtained from each literate patient. Illiterate patients gave oral consent, and then a literate witness sign on behalf of these participants who also indicate their agreement by including their thumbprint on the informed consent form. Non-Lebanese patients were excluded from this study.

### Sample collection and serological tests

Blood samples (2–3 ml) were obtained from each patient before the HD session. Sera were immediately separated, coded and kept frozen at -20°C until used. Samples were then tested for the presence of IgG Antibodies against HEV using a commercially available 96-well plate ELISA kit (Anti-Hepatitis E Virus ELISA IgG, Euroimmun, Lübeck, Germany) showing a specificity and a sensitivity of 100% according to the manufacturer.

### Statistical analysis

Data analysis was performed with Stata 14.2 software. Results were presented as the mean ± standard deviation for quantitative variables and number (percentages) for qualitative variables. Testing for an association between the presence of anti-HEV IgG antibodies and nominal or dichotomous variables was done using the Chi-Square test or Fisher’s exact test where appropriate. To investigate the association between our outcome of interest and the ordinal variables (age, educational level, time since onset of hemodialysis), we used an extension of the Wilcoxon rank-sum test [16]. In order to conclude on the direction of a significant trend, the Somer’s D measure of association was calculated. Somer’s D ranges between -1 and 1, where negative values indicate a negative trend and positive values indicate a positive trend. When treating age as a continuous variable, independent samples t-test was used to assess the presence of group effect. As for the time of onset of HD, the t-test cannot be performed as the variable doesn’t follow a normal distribution. Alternatively, we conducted the non-parametric counterpart which is the Mann-Whitney U (Wilcoxon rank-sum) test. Associations were considered significant at P<0.05.

## Results

Overall, 171 HD patients (96 women and 75 men) participated in this study and were screened for the presence of anti-HEV IgG antibodies in their sera. The mean age of participants was 53.35 ±12.49 years; (age range 23–82). Seropositivity for anti-HEV IgG antibodies was observed in 37 out of 171 patients showing a prevalence rate of 21.63%. Seropositive patients were 22 males and 15 females (male-to-female ratio = 1.46/1) and their mean age was 60.54 ±8.52 years (age range 41–82). As shown in Table 1, statistical analysis demonstrated no significant relationship between HEV infection and gender, education, type of drinking water, history of HBV or HCV infections, the onset of starting HD and the weekly frequency or duration of this latter while treating all the aforementioned variables as categorical (P > 0.05).

**Table 1:** Bivariate analysis of the presence of anti-HEV IgG antibodies among HD patients according to their socio-demographic and clinical characteristics.

Variable	No. (%)	Anti-HEV IgG^+^ n = 37 (21.6%)	Anti-HEV IgG^-^ n = 134 (78.36%)	*P-Value*
**Gender**				0.646
** Male**	96 (56.14)	22 (22.92)	74 (77.08))	
** Female**	75 (43.85)	15 (20)	60 (80)	
**Age**				0.001**
** ≤40**	28 (16.37)	0 (0)	28 (100)	
** 41–60**	89 (52.04)	19 (21.35)	70 (78.65)	
** >60**	54 (31.57)	18 (33.33)	36 (66.67)	
**Education**				0.069
** Illiterate**	43 (25.14)	12 (27.91)	31 (72.09)	
** Primary**	55 (32.16)	14 (25.45)	41 (74.55)	
** Intermediate**	48 (28.07)	8 (16.67)	40 (83.33)	
** Secondary**	15 (8.77)	2 (13.33)	13 (86.67)	
** Tertiary**	10 (5.84)	1 (10)	9 (90)	
**Type of drinking water**				0.482
** Bottled water**	91 (53.21)	18 (19.78)	73 (80.22)	
** Tap water**	72 (42.10)	16 (22.22)	56 (77.78)	
** Spring and well water**	8 (4.67)	3 (37.5)	5 (62.5)	
**HBV infection**				1.000
** Positive**	2 (1.16)	0 (0)	2 (100)	
** Negative**	169 (98.83)	37 (21.89)	132 (78.11)	
**HCV infection**				0.205
** Positive**	4 (2.33)	2 (50)	2 (50)	
** Negative**	167 (97.66)	35 (20.96)	132 (79.04)	
**Onset of starting HD (Years)**				0.446
** ≤5**	97 (56.72)	23 (23.71)	74 (76.29)	
** 6–10**	34 (19.88)	7 (20.59)	27 (79.41)	
** 11–15**	26 (15.20)	4 (15.38)	22 (84.62)	
** 16–20**	5 (2.92)	2 (40)	3 (60)	
** >20**	9 (5.26)	1 (11.11)	8 (88.89)	
**HD Frequency (times per week)**				0.329
** <3**	30 (17.54)	4 (13.33)	26 (86.67)	
** ≥3**	141 (82.45)	33 (23.4)	108 (76.6)	
**HD duration (hours per week)**				0.543
** <5**	113 (66.08)	26 (23.01)	87 (76.99)	
** ≥5**	58 (33.91)	11 (18.97)	47 (81.03)	

** **Indicating statistical significance at 95% confidence level**

In contrast, the age of patients was significantly associated with HEV seropositivity when treated as an ordinal categorical variable as per Table 1 (P = 0.001). The Somer’s D = 0.32 with a p-value = 0.00, showing a significant positive linear trend between the two variables. This shows that the risk of seropositivity of HEV increases in a linear manner with age groups. Similarly, the result of the t-test conducted while treating age as a continuous variable yields a p-value = 0.0001, showing a significant mean difference between HEV positive and negative groups. Specifically, the HEV positive individuals are, on average, 9 years older than those in the HEV negative group.

## Discussion

HEV prevalence among HD patients was previously reported in several Mediterranean countries (Table 2). In contrast, in Lebanon, while recent data concerning the prevalence of hepatitis B and C viruses among national HD patients are available [17], there is no data regarding HEV epidemiology in this population. Thus, our study is the first national report dealing with this subject.

**Table 2:** Prevalence of anti-HEV antibodies among HD patients in Mediterranean countries assessed by ELISA.

Country	Publication Year	Studied sample (n)	% of Seroprevalence	Kit manufacturer	Reference
Greece	1996	420	6.4(IgG); 0(IgM)	Abbott Laboratories	[18]
	1998	211	3.8(IgG); 0(IgM)	Abbott GmbH Diagnostika	[19]
	2004	351	4.8(IgG)	Abbott Diagnostika and Genelabs Diagnostics	[20]
Italy	1996	193	9.3(IgG)	Abbott Laboratories and Nuclear Laser Medicine	[21]
	1997	204	3(IgG)	Abbott Laboratories	[22]
	2014	104	9.6(IgG); 1.9(IgM)	Dia.Pro, Diagnostic BioProbes	[23]
	2015	231	6(IgG/IgM)	Dia.Pro, Diagnostic BioProbes	[24]
	2016	88	25(IgG); 4.5(IgM); 4.5 (IgA)	Wantai and Dia.Pro	[25]
Spain	1995	50	6(IgG); 0(IgM)	Abbott HEV EIA	[26]
	1998	63	6.3(IgG); 0(IgM)	Abbott Laboratories	[27]
Turkey	1996	72	13.9(IgG)	Abbott	[28]
	2009	92	20.6(IgG)	Dia.Pro, Diagnostic BioProbes	[29]
France	1994	147	10.88 (NS); 0(IgM)	Abbott	[30]
Egypt	2015	96	22.9(IgG)	Diagnostic System Italy	[31]
Tunisia	2015	286	10.2(IgG)	Globe Diagnostics Srl	[32]

NS: not specified

Our results showed that among 171 examined patients attending HD units in three different Lebanese hospitals, 37 (21.63%) were positive for anti-HEV IgG antibodies. This rate is higher than those reported in Greece (4.8%) [20], Spain (6.3%) [27] and Tunisia (10.2%) [32], while it is comparable to others reported from Turkey (20.6%) [29] and Egypt (22.9%) [31]. Nevertheless, higher prevalence rates among HD patients were detected in other countries including Iran (28.3%) [12] and England (36.8%) [33].

Previously published data concerning the epidemiology of HEV infection in Lebanon is limited to a sole study in a population of 100 healthy blood donors in which 4% of tested samples were positive for anti-HEV IgG antibodies [34]. More recently, we investigated the serological prevalence of these antibodies among a representative sample of 450 Lebanese pregnant women in Tripoli and found a striking very low prevalence of positivity (0.22%) in this population [35]. Importantly, in contrast to these two studies carried out in healthy populations, our present results reveal a high seroprevalence (21.63%) of anti-HEV IgG antibodies among HD patients. However, it is important to note that the pregnant women population we previously studied, using the same HEV ELISA kit used in this study, is not a suitable age-matched control group that can be compared with the HD patients studied here (mean age 28.33 ± 5.82 years vs. 53.35 ±12.49 years, respectively).

Conflicting results concerning HEV prevalence among chronic HD patients were previously reported. Several reports indicated that HEV infection is more prevalent in this population compared to healthy subjects [12,13,19,30,36,37]. In Isfahan-Iran, Alavian *et al*. detected anti-HEV IgG antibodies in 28.3% of HD patients compared to 9.9% in their control group [12]. Similarly, significantly higher seroprevalence was detected in Argentinian patients undergoing dialysis compared with healthy controls (10.2% and 4.3% respectively) [36]. A report from England also highlights a significantly higher seroprevalence of anti-HEV IgG in HD patients (36.8%) compared to controls (18.8%) [33]. However, other reports showed that HEV prevalence is not significantly higher in HD patients than in other healthy populations. In this context, Sylvan *et al*. previously reported no significant difference between HEV antibodies in Swedish HD patients (6%) and control subjects (5.2%) and no IgG anti-HEV seroconversion during chronic HD [38]. An additional report from Iran also did not found statistically significant higher anti-HEV IgG prevalence among patients with chronic HD [39].

Through which routes HEV is transmitted is a matter of debate. Several reports suggested that oral-fecal transmission is not the sole route of transmission. For example, it was shown that zoonotic, food-borne, person-to-person, vertical, parenteral and nosocomial routes are possible ways of HEV transmission [40]. Possible parenteral transmission of this virus was previously suggested by some studies reporting acute cases of transfusion-transmitted HEV infections and detecting markers of acute infection, including anti-HEV IgM antibodies and HEV RNA in the blood of infected individuals [41–44]. Moreover, these reports found a complete homology of the HEV nucleotide sequences among the blood donor and recipient, and phylogenetic analyses showed that donor–patient HEV isolates clustered together in each case [41,42,44]. A report by Mitsui *et al* also showed that an HD patient became positive for HEV RNA by transfusion of HEV-viremic blood one month after HD initiation, and found identical HEV isolates in the patient’s serum and in that of the transfused viremic blood [45]. In the same way, occasional nosocomial hepatitis E outbreaks were previously reported [46,47], and parenteral HEV transmission may underline such cases. In this context, Siddiqui e*t al*. reported a hospital-acquired outbreak of HEV with a possible parenteral transmission in a neurosurgery ward in Karachi, Pakistan. In this latter report, it was shown that infected patients had IgM antibodies to HEV in their sera and the inappropriate practice of administering mannitol and dexamethasone to patients via shared intravenous administration sets was found as a significant infection risk factor [47].

Overall, the data mentioned above are in favor of a possible parenteral and/or nosocomial HEV transmission. However, in the present study, we don’t investigate markers of acute HEV infection (anti-HEV IgM antibodies or HEV RNA). Also, our results showed that HEV seropositivity was not associated with any HD-related variable including its onset of starting, duration and frequency. In contrast, age was the only parameter associated with HEV seropositivity (Table 1) and the positivity of the Somer’s D measure of association revealed that as age increases, the risk of HEV seropositivity increases. Indeed, while no one subject aged less than 40 years tested positive for anti-HEV IgG antibodies, 21.35% and 33.33% of individuals respectively aged 41–60 and more than 60 years were found seropositive. Our results are in line with those reported in two Swedish [38] and Japanese [48] studies examining HD patients and showing that the prevalence of anti-HEV IgG antibodies increases with age and was markedly higher in patients older than 40 years. In the same way, using logistic regression models, Psichogiou *et al*. [18] and, more recently, Scotto *et al*. [24] found that age was the only variable that presents a statistically significant association with the presence of anti-HEV antibodies in Cypriot and Italian HD patients, respectively. Hence, as suggested in such similar uncontrolled studies, it is possible that the high anti-HEV IgG seroprevalence in our HD population maybe not dependent on the population type but is, rather, due to a possible confounding effect of age. Indeed, this correlation of HEV with older age may reflect a cohort phenomenon possibly due to old infections acquired some decades ago when national sanitation and hygiene conditions were poor and the use of filtered and bottled water was very scarce, even absent, thus promoting the fecal-oral transmission and waterborne spread of HEV.

It is important to note that this study has several limitations. First, we may have missed possible ongoing asymptomatic HEV infections among the enrolled HD patients as we only examined IgG but not IgM antibodies. Second, whilst we use the presence of anti-HEV IgG antibodies in HD sera as an infection marker, it is important to note that available serological assays used to detect these antibodies show great variability in their sensitivity [6,7] as revealed in several previous studies [49,50]. To note, in a comparative study analyzing the performance of five ELISAs for anti-HEV antibodies, the one used in the present study showed the lowest reactivity [50]. Overall, this may make the estimation of reliable seroprevalence in our study population challenging. Third, we don’t investigate the proportion of HD patients who receive blood transfusions which is a common possible feature in this population and is also a possible risk factor of HEV seropositivity. Finally, the study lacks an age-matched control group of healthy individuals which is a needed feature to identify if HD patients are at higher risk of HEV infection than their control counterparts and, if so, determine the reasons behind such increased risk.

In conclusion, for the first time, we reported that almost the one-fifths of HD patients in Tripoli, Lebanon tested positive for anti-HEV IgG antibodies. Our data reflect an important HEV prevalence in this group. Effective health strategies are therefore required to prevent this HEV circulation potential among HD patients. However, further studies, enrolling sex- and age-matched controls, with larger sample sizes and from different Lebanese geographical areas are needed to confirm our findings and clarify the exact transmission route of HEV in HD patients.
